# Effects of Vitamin D Intake on FEV1 and COPD Exacerbation: A Randomized Clinical Trial Study

**DOI:** 10.5539/gjhs.v7n4p243

**Published:** 2015-01-14

**Authors:** Abolfazl Zendedel, Mohammadreza Gholami, Khatereh Anbari, Kourosh Ghanadi, Elham Ceneicel Bachari, Alireza Azargon

**Affiliations:** 1 School of Medicine, Lorestan University of Medical Sciences, Khorramabad, Iran

**Keywords:** COPD, Exacerbation, FEV1, Vitamin D

## Abstract

**Aim:**

This study aimed to evaluate the effects of vitamin D intake on COPD exacerbation and FEV1 in the patients with severe and very severe COPD.

**Methods:**

This double blind placebo control randomized clinical trial study was done in the Ashayer university hospital in Khorramabad in 2012. Eighty eight patients with severe and very severe COPD were randomly selected from those who recoursed to the internal medicine clinic of Ashayer hospital. They were randomly allocated to case and placebo group. The patients received routine treatment for COPD. Along with the routine treatment, placebo group received 100,000 IU of oral vitamin D per month, for 6 months. Data was analyzed using SPSS computer software, paired t-test, independent t-test, non parametric t-test and Pearson correlation coefficients.

**Results:**

In each group, there were 44 patients. After the intervention, there were significant differences in FEV1 and the number of COPD exacerbation between the case and control group patients. Also, after the study, in the case group, FEV1 was increased and the number of COPD exacerbation was decreased significantly.

**Conclusion:**

Vitamin D intake decreased COPD exacerbation and improved FEV1 in the patients with severe and very severe COPD. It is suggested that baseline serum vitamin D levels will recorded in similar studies and the effect of vitamin D intake will evaluated regarding the baseline serum vitamin D levels.

## 1. Introduction

Chronic Obstructive Pulmonary Disease (COPD) is a chronic disease that causes persistent airflow obstruction. The airflow obstruction in this disease is generally progressive (MacNee et al., 2005). COPD has two clinical phases (stable phase and exacerbation phase), both of which are associated with inflammation (Barbu et al., 2011). Smoking, passive smoking, reactivity of airways, occupational factors and air pollution are the risk factors of COPD (Reilly et al., 2004). Independent risk factors for COPD are male gender, advanced age, low socioeconomic status, occupational exposure and cigarette smoking (Caballero et al., 2008).

Based on the World Health Organization estimation, COPD will be the third cause of mortality in the world in 2020 (Murray et al., 1997). Ninety percent of COPD deaths occur in low and middle income countries (Murray et al., 1997). In European countries, depending on the age of participants, the methods used and the location, the prevalence of COPD ranged from 2.1% to 26.1% (Atsou et al., 2011). It was 8.9% in India (from 6.2% to 13.5%; based on spirometry) (Afonso et al., 2011), 3.02% in the Netherlands (in a population-based study including subjects ≥ 40) (8), 17.4% in Copenhagen (aged 35 years or older) (Fabricius et al., 2011) and 3.7% in Abu Dhabi (in 40-80 year old subjects) (Al Zaabi et al., 2011).

Nowadays, the attention to nonskeletal effects of vitamin D has been increased (Kunisaki et al., 2011). An association between pulmonary function and serum vitamin D levels has been reported in some studies. It has been reported that vitamin D deficiency correlates with severity of COPD (Janssens et al., 2010). Also, in some studies, it has been declared that COPD patients had a raised risk for vitamin D deficiency (Persson et al., 2012, Zhang et al., 2012). Likewise, in one study, it has been stated that total vitamin D intake was negatively associated with COPD (Shaheen et al., 2011).

According to previous studies, the effect of levels vitamin D is controversial on COPD exacerbation and FEV1. This study aimed to evaluate the effect of vitamin D intake on COPD exacerbation and FEV1 in the patients with severe and very severe COPD.

## 2. Method

This double-blind, placebo-controlled, randomized clinical trial was done in Ashayer university hospital in Khorramabad in 2012. The Ethics and Research Committee of Lorestan University of Medical Sciences approved this study. Furthermore, we obtained signed informed consents from all the participants. This study has been recorded in Iranian Registry of Clinical Trials at www.irct.ir as a clinical trial (IRCT2012071810332N1). Eighty-eight patients with severe and very severe COPD were randomly selected from those who recoursed to the internal medicine clinic of Ashayer hospital. Severe and very severe COPD were defined as Global Initiative for Chronic Obstructive Lung Disease (GOLD) guidelines (Donaldson et al., 2002). The selected patients were allocated to the study and placebo groups by simple random sampling method. The patients in both the groups received the routine treatment for COPD. Along with the routine treatment, the study group received 100,000 IU of oral vitamin D per month, for 6 months. In contrast, the placebo group received oral placebo for 6 months. Before the study, forced expiratory volume in 1 second (FEV1) was determined, and the number of COPD exacerbations during the last 6 months was recorded in the both groups. After 6 months of treatment, FEV1 was determined, and the number of COPD exacerbations during the study span was evaluated in the both groups. The patients received a telephone call every 2 months to assess respiratory symptoms consistent with a COPD exacerbation. The definition of COPD exacerbation was either the presence of 2 or more of these major symptoms (increase in sputum purulence, sputum volume or dyspnea) or any of major symptoms accompanied by any of minor symptoms (increase in nasal discharge, wheeze, sore throat, cough or fever) for at least two consecutive days (Donaldson et al., 2002).

### 2.1 Statistics

The data was analyzed using the SPSS computer software and paired t-test, independent t-test, non-parametric t-test and Pearson correlation coefficients. The P-values<0.05 were considered statistically significant.

## 3. Results

In each group, there were 44 patients, 30 of whom (68.2%) were male. There were no significant differences between age distribution, cigarette smoking and addiction in the study and placebo groups (Table 1).

**Table 1:** Age distribution, cigarette smoking and addiction in case and placebo groups

		Case group Number (%)	placebo group Number (%)	p value
Age (Year)	<45	2 (4.5%)	1(2.3%)	0.83
	45-60	15 (34.1%)	16 (36.4%)	
	>60	27 (61.4%)	27 (61.4%)	
Cigarette smoking (cigarette per month)	None	11 (25%)	13 (29.5%)	0.22
	< 50	26 (59.1%)	23 (52.3%)	
	> 50	7 (15.9%)	6 (13.6%)	
Addiction	None	17 (38.6%)	18 (40.9%)	0.53
	Oral use	9 (20.4%)	8 (18.2%)	
	Inhalation use	16 (36.4%)	18 (40.9%)	
	Injection use	2 (4.5%)	0	

The mean of FEV1 and COPD exacerbations according to sex, age distribution, cigarette smoking and addiction, before and after the study, are shown in Table 2 and Table 3.

**Table 2:** The mean of FEV1 according to sex, age distribution, cigarette smoking and addiction, before and after the study

		Case			placebo		

		Before	After	p value	Before	After	p value
Sex	Male	33.6±7.7	51.2±5.9	0.0001	33.6±4.6	33.1±9.7	0.44
	Female	36.8±10	52.5±14.6	0.009	35±8.6	34.2±4.6	0.32
	p value	0.3	0.76	-	0.17	0.59	-
Age (Year)	<45	39±1.4	55±4.2	-	35±0	32	-
	45-60	36.8±8	51±13.9	0.004	35.6±9.4	34.2±8.4	0.062
	>60	33.1±8.9	51.7±6.4	0.0001	34.5±9.2	34.8±7.7	0.13
	p value	0.3	0.85	-	0.88	0.53	-
Cigarettesmoking (cigarette/month)	None	35.2±8.4	49.3±15.6	0.047	38.7±9.3	34.1±6.7	0.018
	< 50	39.5±3.6	55.7±5.1	<0.0001	38.7±4.4	35.7±5.4	0.011
	> 50	33.07±9.1	51.5±6.4	<0.0001	32.1±9.2	31.1±8.7	0.32
	p value	0.19	0.38	-	0.063	0.31	-
Addiction	None	37±8.6	51.1±13.3	0.003	38.3±6.2	34.4±6.1	0.001
	Oral use	37.1±7.6	54.6±5.1	<0.0001	36.1±11.7	30±9.2	0.015
	Inhalation use	32.1±7.8	51.6±6.7	<0.0001	32.6±9.7	30.2±8	0.078
	Injection use	34±9.8	42.5±3.5	-	-	-	-
	p value	0.187	0.42	-	0.164	0.23	-

**Table 3:** The mean of COPD exacerbation according to sex, age distribution, cigarette smoking and addiction, before and after the study

		Case			Control		

		Before	After	p value	Before	After	p value
Sex	Male	18.8±3.5	9.8±1.3	0.0001	19.3±4.2	19.6±3.9	0.056
	Female	16.3±2.4	9.3±1.3	0.0023	18.1±2.5	17.7±2.5	0.73
	p value	0.095	0.25	-	0.23	0.053	-
Age (Year)	<45	19±1.4	10	-	18±0	18	-
	45-60	16.8±3.3	9.5±1.1	0.0001	18.8±1.4	17.8±1.6	0.79
	>60	18.2±2.8	9.7±1.5	<0.0001	19.4±2.9	19.7±2.9	0.64
	p value	0.28	0.82	-	0.66	0.069	-
Cigarette smoking (cigarette/month)	None	17.1±1.9	8.8±1.3	<0.0001	17.3±2.6	17.1±2.3	0.65
	< 50	18.5±2.2	9.8±1.4	<0.0001	19.2±3.3	19.2±2.8	0.38
	> 50	19.1±3.4	10±1.2	<0.0001	19.1±4.4	19.4±4.1	0.89
	p value	0.185	0.038	-	0.42	0.2	-
Addiction	None	16.1±2.8	9.2±1.2	<0.0001	17.5±3.1	17.8±3.5	0.42
	Oral use	20.4±4.7	10±1.8	<0.0001	20±3.7	20.2±3.9	0.56
	Inhalation use	18.9±1.7	10±1.1	<0.0001	19.2±4.4	19.2±3.4	0.99
	Injection use	15±2.1	10±1.4	-	-	-	-
	p value	0.023	0.35	-	0.24	0.24	-

Before the study, there were no significant differences in FEV1 and the number of COPD exacerbations between the study and placebo group patients. But, after the study, there were significant differences in FEV1 and the number of COPD exacerbations between the study and placebo group patients. Also, after the study, in the study group, FEV1 was increased and the number of COPD exacerbations was decreased significantly (Table 4).

**Table 4:** FEV1 and the number of COPD exacerbation in the case and placebo groups

		Case	Control	p value
FEV1 (M±SD)	Before	34.6±8.5	34.4±9.2	0.89
	After	51.6±9.4	31.9±7.6	<0.001
	p value	<0.001	0.53	-
COPD exacerbation (M±SD)	Before	18.02±3.3	18.7±3.8	0.38
	After	9.7±1.3	18.8±3.6	<0.001
	p value	<0.001	0.83	-

## 4. Discussion

COPD is a chronic and common disease. COPD can cause severe complications. Afonso et al., reported that 26% and 2.8% of the patients with very severe COPD and non-COPD patients had died after 1 year of follow-up in the Netherlands (Afonso et al., 2011).

An association between pulmonary function and serum vitamin D levels has been reported in some studies. It has been reported that vitamin D deficiency correlates with the severity of COPD (Janssens et al., 2010). Also, it has been reported that a significant relation between FEV1 and serum 25-hydroxy vitamin D levels (Azargoon et al., 2011). However, in a study, baseline 25-hydroxy vitamin D levels were not predictive of acute exacerbation in patients with severe COPD (Kunisaki et al., 2012). But the relationship between vitamin D and COPD has been reported in some studies. Also, it has been stated that total vitamin D intake was negatively associated with COPD (Shaheen et al., 2011). Regarding these results, vitamin D intake can be beneficial in COPD patients. This study aimed to evaluate the effect of vitamin D intake on COPD exacerbation and FEV1 in the patients with severe and very severe COPD. According to our knowledge, the effect of vitamin D on FEV1 and COPD exacerbations has been studied in few studies.

In this study, vitamin D intake improved COPD exacerbations and FEV1 in the patients with severe and very severe COPD. Hornikx et al., reported that 100.000 IU of vitamin D per month for one year had improved maximal oxygen uptake and inspiratory muscle strength significantly in the COPD subjects who had followed a rehabilitation program (Hornikx et al., 2012). These findings can vindicate the results of our study.

In a similar study, Lehouck et al., compared the effects of vitamin D and placebo on FEV1 and exacerbation rate in the patients with moderate to very severe COPD (Lehouck et al., 2012). In their study, each patient received 100,000 IU of vitamin D every 4 weeks for 1 year. In contrast to our results, they reported that this dose of vitamin D had not improved FEV1 and exacerbation rate. This difference may be due to the difference between baseline serum vitamin D levels in these studies. Although serum vitamin D level was not determined in our study, some studies have stated that vitamin D deficiency is prevalent in Iran. Studies suggest that Vitamin D increase production IL-10, an antiinflammatory cytokine involved in the pathogenesis of asthma, from T cells, increase production IL-37, antimicrobial peptide, regulate matrix metalloproteinases (MMP), shifting theTh1 and Th2 balance and reducing inflammation (Xystrakis et al., 2006, De Smet et al., 2005; Finklea et al., 2011). Taken together vitamin D intake (100,000 IU per 4 weeks for 6 months) improved COPD exacerbation and FEV1 in the patients with severe and very severe COPD significantly. It is suggested that baseline serum vitamin D levels be recorded in similar studies and the effect of vitamin D intake be evaluated regarding the baseline serum vitamin D levels.
